# Development of 1,2,3-Triazole-Based Sphingosine Kinase Inhibitors and Their Evaluation as Antiproliferative Agents

**DOI:** 10.3390/ijms18112332

**Published:** 2017-11-05

**Authors:** Angela Corvino, Roberta Rosa, Giuseppina Maria Incisivo, Ferdinando Fiorino, Francesco Frecentese, Elisa Magli, Elisa Perissutti, Irene Saccone, Vincenzo Santagada, Giuseppe Cirino, Maria Antonietta Riemma, Piero A. Temussi, Paola Ciciola, Roberto Bianco, Giuseppe Caliendo, Fiorentina Roviezzo, Beatrice Severino

**Affiliations:** 1 Department of Pharmacy, School of Medicine, University of Naples Federico II, 80131 Naples, Italy; angela.corvino@unina.it (A.C.); giuseppinamaria.incisivo@unina.it (G.M.I.); fefiorin@unina.it (F.F.); frecente@unina.it (F.F.); elisa.magli@unina.it (E.M.); elisa.perissutti@unina.it (E.P.); irene.saccone@unina.it (I.S.); santagad@unina.it (V.S.); cirino@unina.it (G.C.); mariaantonietta.riemma@unina.it (M.A.R.); caliendo@unina.it (G.C.); roviezzo@unina.it (F.R.); 2 Department of Clinical Medicine and Surgery, Oncology Division, University of Naples Federico II, 80131 Naples, Italy; roberta.rosa@unina.it (R.R.); paola.ciciola@unina.it (P.C.); robianco@unina.it (R.B.); 3 The Wohl Institute, King’s College London, 5 Cutcombe Rd, London SE5 9RT, UK; temussi@unina.it

**Keywords:** Sphingosine Kinase 1, Sphingosine Kinase 2, inhibitors, synthesis, antiproliferative activity

## Abstract

Two series of *N*-(aryl)-1-(hydroxyalkyl)pyrrolidine-2-carboxamides (**2a**–**2g** and **3a**–**3g**) and 1,4-disubstituted 1,2,3-triazoles (**5a**–**5h** and **8a**–**8h**) were synthesized. All the compounds, containing a lipophilic tail and a polar headgroup, were evaluated as sphingosine kinase (SphK) inhibitors by assessing their ability to interfere with the acetylcholine (Ach) induced relaxation of aortic rings pre-contracted with phenylephrine. Moreover, their antiproliferative activity was tested on several cell lines expressing both SphK1 and SphK2. Compounds **5h** and **8f**, identified as the most efficient antiproliferative agents, showed a different selectivity profile, with **8f** being selective for SphK1.

## 1. Introduction

Two isoforms of sphingosine kinase have been identified (SphK1 and SphK2) [1]. Although they exhibit subtle differences in the substrate specificity and subcellular localization, SphK1 and SphK2 catalyze the same reaction, i.e., phosphorylation of sphingosine to produce sphingosine-1-phosphate (S1P).

S1P is a bioactive lipid that activates a family of G protein-coupled receptors, termed S1P1–5. SphK1 is mostly localized to the cytosol while SphK2 is mostly nuclear [2]. SphK1 and SphK2 double knockout mice exhibit a severely disturbed neurogenesis and angiogenesis, causing embryonic lethality [3]. Nonetheless, mice knocked out for just one of the two kinases are viable, fertile, and without any obvious abnormalities, mainly because of some compensatory activities of the two enzymes [4,5].

The S1P pathway has a pivotal role in the control of immune cell trafficking and has been involved in inflammatory based diseases and cancer [6,7,8]. Up-regulation of SphK1 has been found in a wide array of human solid cancers and hematological malignancies [9]; the consequent elevated cellular levels of S1P promote cell survival, proliferation [10], and angiogenesis, indicating a major implication for SphK1 in the development and progression of cancer and chemo-resistance [11]. Nonetheless, high potency SphK1 inhibitors have shown effects on S1P levels but not on cell viability; moreover, most of them have shown several off-target activities.

The potential of SphK2 as an oncology target has gained increasing interest. SphK2 can be considered as a prognostic negative index in non-small cell lung cancer [12]. Moreover, small interfering RNA (siRNA) knockdown studies have indicated that this isoform has a crucial role in several cancers, including acute lymphocytic leukaemia, multiple myeloma, glioblastoma, kidney, and breast cancer [13].

Experiments performed knocking down SphK1 and SphK2 suggest the existence of a compensatory mechanism for the two isoforms. Thus, several research groups have focused their attention on the development of dual inhibitors to study this phenomenon further [14,15]. On the other hand, the hunt for selective SphK1 and SphK2 inhibitors is crucial in elucidating the specific functions of the two isoforms and has led to the description of a plethora of molecules, most of them targeting the sphingosine-binding pocket of the enzyme [13,15,16]. The resolution of the SphK1 crystal structure [17] has given a boost to the discovery of novel SphK inhibitors, recently leading to the identification of potent isoform selective inhibitors. The inhibitors have been instrumental in elucidating the roles of SphKs in the regulation of key oncogenes and their involvement in inflammatory signaling. However, the most commonly used inhibitors failed to induce cancer cell death [18]; moreover, important off-targets have been recently reported [19,20].

**Scheme 1:**
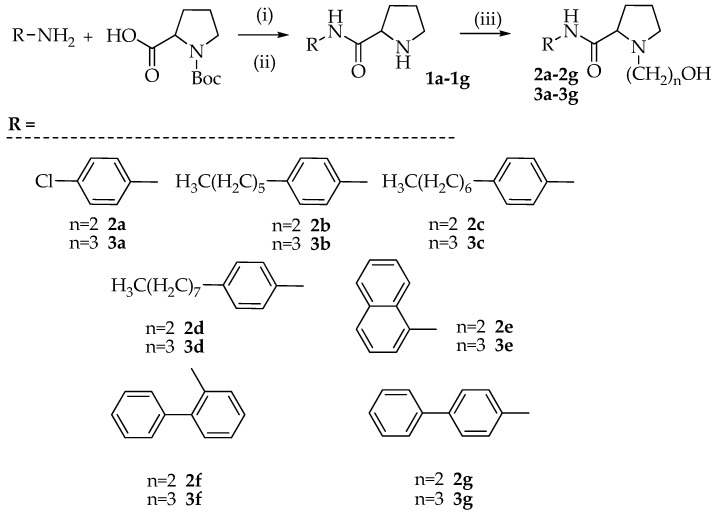
Reagents and conditions: (i) TBTU/HOBt, dimethylformamide (DMF), 12 h; (ii) 40% trifluoroacetic acid (TFA) in DCM, 2 h; (iii) Br(CH_2_)_n_OH, NaI, K_2_CO_3_, DMF, 12 h.

Starting from these considerations and bearing in mind the need of SphK inhibitors to increase the possibility of having useful new therapeutic agents, we designed a first series of *N*-(aryl)-1-(hydroxyalkyl)pyrrolidine-2-carboxamides (compounds **2a**–**2g** and **3a**–**3g**; Scheme 1).

The newly synthesized molecules, possessing some structural elements in common with already known inhibitors, are characterized by the presence of a polar head linked through a pyrrolidine spacer to a lipophilic tail, represented by several aromatic moieties, such as 4-chlorophenyl, 4-hexylphenyl, 4-heptylphenyl, 4-octylphenyl, α-naphthyl, 2-biphenyl, and 4-biphenyl nuclei. All the compounds were routinely evaluated by first assessing their ability to interfere with the acetylcholine (Ach) induced relaxation of aortic rings pre-contracted with phenylephrine (PE). Roviezzo et al. have demonstrated that incubation of rat aorta rings with a SphK inhibitor produced a concentration-dependent reduction of Ach-induced vasorelaxation [21]. This experiment revealed that only the 4-octylphenyl derivatives **2d** and **3d**, at the tested concentration of 30 µM, were able to inhibit the aortic ring relaxation. This nucleus was selected for the development of two series of triazole derivatives, the more rigid **5a**–**5h** and the more flexible **8a**–**8h**. The compounds were firstly evaluated on aortic rings, and then tested for their antiproliferative ability on several cell lines expressing both SphK1 and SphK2.

## 2. Results and Discussion

### 2.1. Synthesis

The synthesis of compounds **2a**–**2g** and **3a**–**3g** was accomplished in three steps as outlined in Scheme 1.

Intermediates **1a**–**1g** were prepared by reaction of the opportune aromatic amine with Boc-Pro-OH via 2-(1H-benzotriazole-1-yl)-1,1,3,3-tetramethylaminium tetrafluoroborate/1-hydroxybenzotriazole hydrate (TBTU/HOBt) in dimethylformamide (DMF) and subsequent Boc removal with 40% trifluoroacetic acid (TFA) in dichloromethane (DCM) for 2 h. The final compounds **2a**–**2g** and **3a**–**3g** were prepared by reacting the opportune intermediate with 2-bromoethanol or 3-bromopropanol, respectively, in the presence of a 50% excess of K_2_CO_3_ and NaI, in DMF solution. The yields, referred to in the final step, were in the range 54–73% for the ethyl derivatives (**2a**–**2g**) and 52–84% for the propyl derivatives (**3a**–**3g**).

**Scheme 2:**
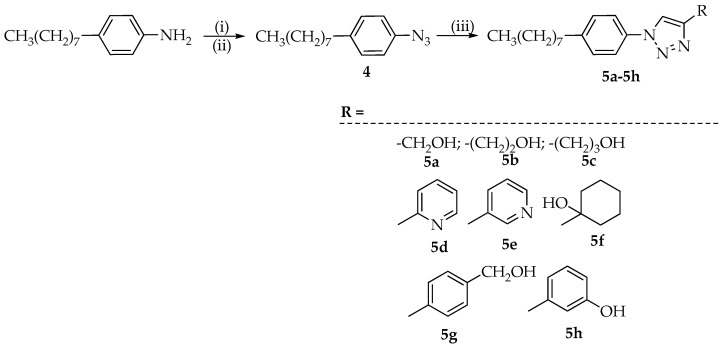
Reagents and conditions: (i) NaNO_2_, HCl conc., −2 °C, 30 min; (ii) NaN_3_, H_2_O, 0 °C, 12 h; (iii) R-alkyne, CuSO_4_, sodium L-ascorbate, H_2_O/tBuOH 1/1 (*v*/*v*), 48 h.

Compounds **5a**–**5h** were prepared according to the procedure depicted in Scheme 2. 4-Octylaniline was converted to the corresponding azide **4** firstly by producing the diazonium salt by reaction with NaNO_2_ in concentrated HCl and subsequently, by treatment with NaN_3_ in water, at 0 °C for 12 h. The following Cu-catalyzed azide-alkyne cycloaddition of **4** with the opportune alkyne, performed using Sharpless protocol [22], afforded the final desired 1,4-disubstituted 1,2,3-triazoles **5a**–**5h** with yields in the range 77–90%.

**Scheme 3:**
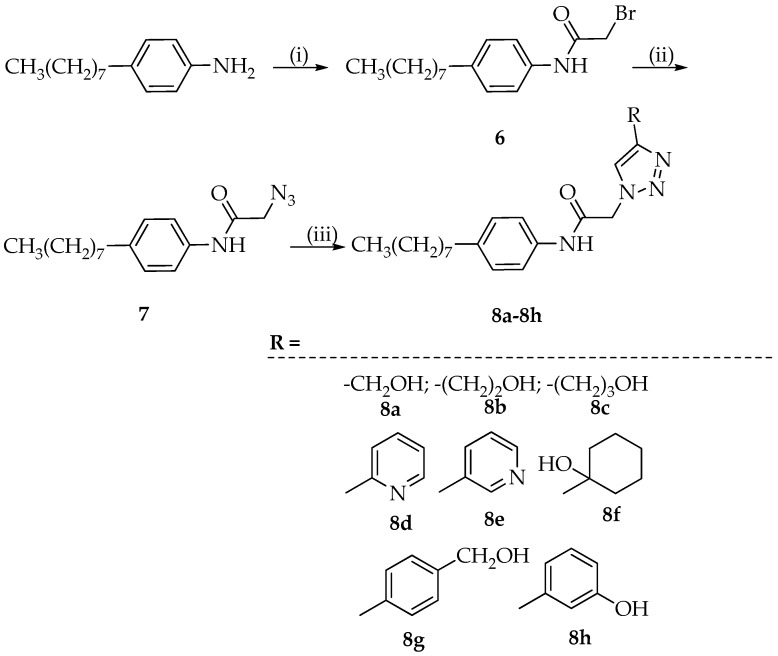
Reagents and conditions: (i) 2-bromoacetyl bromide, toluene, 4 h, 110 °C; (ii) NaN_3_, EtOH, reflux for 2 h; (iii) R-alkyne, CuSO_4_, sodium L-ascorbate, H_2_O/tBuOH 1/1 (*v*/*v*), 48 h.

Finally, compounds **8a**–**8h** were prepared as reported in Scheme 3. Firstly, 4-octylaniline was reacted with 2-bromoacetyl bromide obtaining the intermediate 6. This latter substance, treated with NaN_3_ in ethanol under reflux and followed up with Sharpless protocol, afforded the final compounds **8a**–**8h**, with yields in the range 75–85%.

### 2.2. Aorta Rings Assay

The effect of the potential inhibitors of sphingosine kinases (SphKs) has been evaluated on Ach-induced vasorelaxation in isolated mouse aorta rings. Indeed, the SphK/S1P pathway is involved in Ach-induced relaxation [21]. Aortic rings were incubated with each compound (30 min; 1–30 μM) prior to performing a cumulative dose-response to Ach. Compounds **2d** (53%) and **3d** (60%) significantly inhibited Ach-induced relaxations.

These compounds were liquid and had a very low solubility in aqueous solution. For these reasons, we designed a new series of compounds with the aim to overcome these difficulties. Considering the inhibitory properties of the octylphenyl derivatives **2d** and **3d**, we synthesized two series of triazoles, **5a**–**5h** and **8a**–**8h**, in which the octylphenyl group is linked to a polar head using the triazole nucleus as the linkage element. All the compounds have been tested, as described above, and most of them displayed a significant inhibitory activity at 30 μM (Table 1).

### 2.3. Antiproliferative Activity

We first performed a drug screening by testing the capability of the triazole derivatives **5a**–**5h** and **8a**–**8h** to reduce cell density of cultured A549, H1975, and HCC827 human non-small cell lung cancer (NSCLC) cell lines. The synthesized molecules were divided in three groups by using the dose that reduced cell density by 50% (Table 1). For most of the compounds, the dose able to reduce cell density by 50% was >10 µM; for compounds **5c** and **5f**, this dose was between 5 and 10 µM; only for **5h** and **8f** was it ≤5 µM.

**Figure 1:**
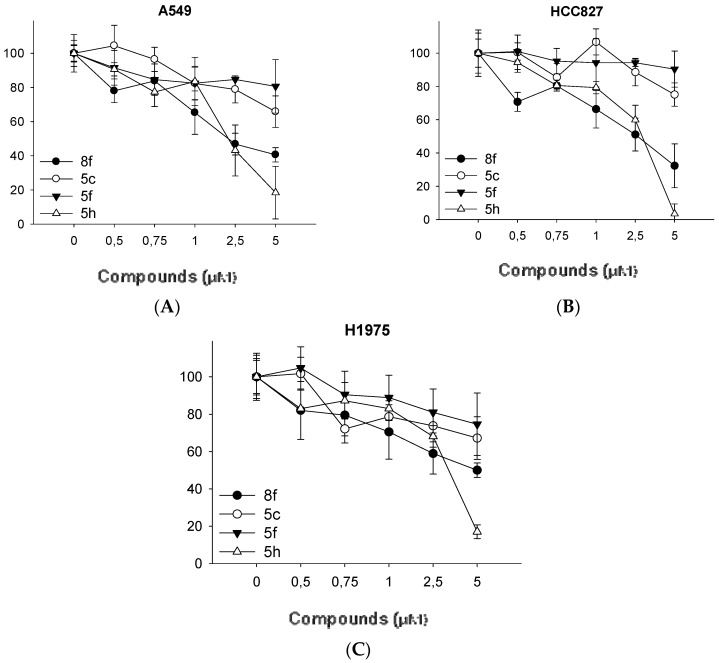
The compounds **5c**, **5f**, **5h**, and **8f** are able to reduce cell density of cultured NSCLC cell lines. Percent of cell density of A549 (**A**), HCC827 (**B**), and H1975 (**C**) NSCLC cells treated with different doses (0.5–5 µM) of the compounds **5c**, **5f**, **5h**, and **8f** for 3 days, as measured by 3-(4,5-dimethylthiazol-2-yl)-2,5-diphenyltetrazolium bromide (MTT) assay. Data represent the mean (±SD) of three independent experiments, each performed in triplicate. Bars show SDs. Statistical significance was determined by the Student *t*-test: *p* < 0.1 for compounds **5c** (5 µM, A549), **5h** (2.5 µM, all cell lines), and **8f** (2.5 µM, all cell lines); *p* < 0.01 for compounds **5h** (5 µM, all cell lines) and **8f** (5 µM, all cell lines).

The four best compounds (**5c**, **5f**, **5h**, and **8f**), were analyzed in a comparative manner by using the same experimental procedure and NSCLC cell lines listed above. Compounds **5h** and **8f** confirmed the results obtained in the drug screening, they were the more efficacious among the synthesized molecules. Both the compounds were able to reduce cell density by 50% or more at the dose of 5 µM (Figure 1) and **5h** was more effective than **8f** on all the tested cell lines with a 100% reduction of cell density on HCC827 cells, when tested at 5 µM.

**Figure 2:**
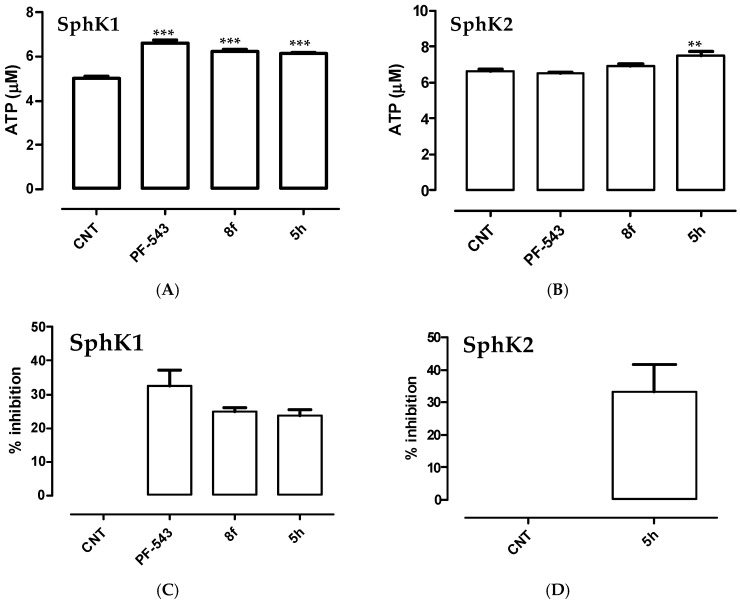
Effect of the tested compounds on the adenosine triphosphate (ATP) levels in the presence of SphK1 and SphK2. (**A**,**B**): ATP concentrations (µM) were measured by the Sphingosine Kinase Activity Assay (Echelon Biosciences) in the presence of SphK1 (**A**) and SphK2 (**B**), and tested with PF-543, **5h**, and **8f**. All the compounds were evaluated at the concentrations of 10 µM. For further details, see the Materials and Methods section. Data represent the mean (±SD) of two experiments performed in triplicate. Bars show SDs. (**C**,**D**): Percent of enzyme inhibition by PF-543, **5h**, and **8f** on SphK1 (**C**) and by **5h** on SphK2 (**D**), respectively, as measured by the Sphingosine Kinase Activity Assay (*Echelon Biosciences*).

To verify the selectivity of **5h** and **8f** towards the two enzymatic isoforms, we performed a SphK activity assay. The principle of the method is to quantify, by luminescence, the remaining amount of adenosine triphosphate (ATP) in solution following the kinase reaction. The luminescent signal is inversely correlated with the kinase activity. The SphK1 competitive inhibitor PF-543 was used as a positive control for SphK1 and as a negative control for SphK2. As shown in Figure 2A, PF-543, **5h**, and **8f** tested at 10 µM, were able to inhibit SphK1 activity as demonstrated by the higher ATP levels in solution when compared to that of the control. When tested on SphK2, only compound **5h** caused an increment of ATP levels, with respect to the control, indicating that it is able to inhibit both the enzymes (Figure 2B).

In order to better display these results, we converted the data to percent of inhibition, obtaining the graphs reported in Figure 2C,D. As displayed, PF-543 was completely ineffective on SphK2 (Figure 2B) and selectively inhibited SphK1 (Figure 2A), even if it was less effective than expected. Moreover, when it was tested on A549 cells, it reduced cell density by less than 50% at 5 µM (Figure S1) showing an antiproliferative effect lower than that of the here reported compounds. Finally, compound **8f** was able to selectively inhibit SphK1, while compound **5h** showed a comparable inhibition of both the enzyme isoforms SphK1 and SphK2. This data proposes **5h** as a dual inhibitor and might explain the higher efficacy of **5h** as an antiproliferative agent. Several groups have reported that knockdown of SphK1 or SphK2 has produced compensatory increases in expression of the other isoform [13]. This phenomenon supports the need for more potent SphK1/SphK2 dual inhibitors.

### 2.4. Specific **8f** Versus Aspecific **5h**

Is there a conceivable intrinsic difference between these two compounds that can rationalize the specificity of **8f** with respect to the SphK1 isoform? In principle, it is very difficult to discuss this problem in terms of conformational properties of the two compounds, mainly because only the structure of the SphK1 isoform is known (PDB: 3VZB), whereas that of the SphK2 isoform is still unknown. However, it is possible to compare the molecular models of **8f** and **5h** in a simple qualitative way and draw a tentative hypothesis.

**Figure 3:**
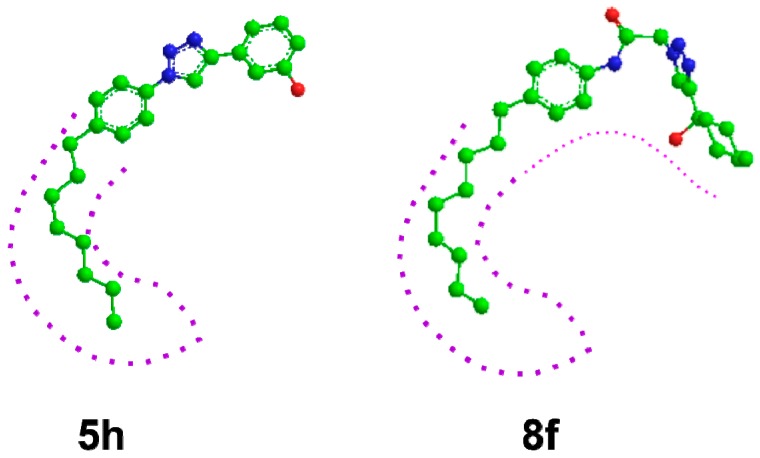
Comparison of the molecular models of inhibitors **5h** and **8f**. The outline of the “J” site, typical of the SphK1 isoform active site is superimposed to both models. In the case of the **8f** model, the outline is prolonged to show the good complementarity with the shape of the active site.

Molecular models of **8f** and **5h** are shown in Figure 3 side by side, superimposed to the silhouette of the “J” site. It is possible to appreciate that the head of **8f** has a conformation complementary to the shape of the active site of the SphK1 isoform, whereas the corresponding moiety of **5h** is simpler (and shorter) and can thus adapt, in principle, to the (presumably) different shapes of SphK1 and SphK2 isoforms.

## 3. Materials and Methods

### 3.1. Chemistry

All reagents and solvents were purchased from Sigma-Aldrich (Milan, Italy). Melting points, determined using a Buchi Melting Point B-540 instrument, are uncorrected and represent values obtained on recrystallized or chromatographically purified material. ^1^H-NMR and ^13^C-NMR spectra were recorded on Varian Mercury Plus 400 MHz instrument. Spectra of compounds **2a**–**2g** and **3a**–**3g** were recorded in CDCl_3_, while those of compounds **5a**–**5h** and **8a**–**8h** were recorded in dimethyl sulfoxide (DMSO)-d_6_. Chemical shifts are reported in ppm. The following abbreviations are used to describe peak patterns when appropriate: s (singlet), d (doublet), t (triplet), m (multiplet), bs (broad singlet), mm (multiplet of multiplet). Mass spectra of the final products were performed on an API 2000 Applied Biosystem mass spectrometer. Elemental analyses were carried out on a Carlo Erba model 1106; analyses indicated by the symbols of the elements were within ±0.4% of the theoretical values. All reactions were followed by thin-layer chromatography (TLC), carried out on Merck silica gel 60 F_254_ plates with a fluorescent indicator, and the plates were visualized with UV light (254 nm). Preparative chromatographic purifications were performed using a silica gel column (Kieselgel 60). Solutions were dried over Na_2_SO_4_ and concentrated with a Buchi R-114 rotary evaporator at low pressure. Homogeneity of the products was assessed by analytical reversed-phase high pressure liquid chromatography (HPLC) using a Macherey-Nagel Nucleosil 100-5 C18 (5 μm, 4 × 125 mm), applying the following gradient of 0.1% (*v*/*v*) trifluoroacetic acid (TFA) in H_2_O (solvent A) and 0.1% TFA (*v*/*v*) in acetonitrile (solvent B) at a constant flow of 1 mL/min: (i) gradient 30–60% B over 20 min. The column was connected to a Rheodyne model 7725 injector, a Waters 600 HPLC system, a Waters 486 tunable absorbance detector set to 220 nm, and a Waters 746 chart recorder.

### 3.2. Synthesis of N-(aryl)-1-(hydroxyalkyl)pyrrolidine-2-carboxamide Derivatives (Compounds **2a**–**2g** and **3a**–**3g**)

#### 3.2.1. Synthesis of *N*-(4-chlorophenyl)pyrrolidine-2-carboxamide (**1a**)

To a solution of Boc-Pro-OH (1 mmol) and *p*-chloro-aniline (1 mmol) dissolved in anhydrous DMF (15 mL) were added TBTU (1.1 mmol), HOBt (1.1 mmol), and *N*, *N*-diisopropylethylamine (DIPEA) (1.1 mmol). The reaction mixture was stirred for 12 h at room temperature. After solvent removal, the residue was dissolved in ethyl acetate and extracted with 10% citric acid, 5% NaHCO_3_, and brine. The organic phase was dried with Na_2_SO_4_, filtered, and evaporated to dryness. The residue was purified on a silica gel column using DCM/MeOH (9.5/0.5 *v*/*v*) as an eluent. The crystallization with diethyl ether provided the Boc protected product that was dissolved in 40% TFA in DCM (10 mL) and the mixture was stirred at room temperature for 2 h. After evaporation of the solvent, the desired product **1a** was obtained as a white solid by crystallization with diethyl ether (yield 47%, calculated on two steps).

Using the above described procedure intermediates **1b**–**1g** were prepared starting from 4-hexylaniline (**1b**), 4-heptylaniline (**1c**), 4-octylaniline (**1d**), α-naphtylamine (**1e**), 2-aminobiphenyl (**1f**), and 4-aminobiphenyl (**1g**).

#### 3.2.2. *N*-(4-chlorophenyl)-1-(2-hydroxyethyl)pyrrolidine-2-carboxamide (**2a**)

A solution of 2-bromoethanol (1 mmol) and NaI (1.5 mmol) dissolved in 15 mL of DMF was stirred under reflux for 30 min. Then, a solution of **1a** (1 mmol) and K_2_CO_3_ (1.5 mmol) in 10 mL of DMF was added drop wise. After 12 h, the reaction mixture was cooled at room temperature, the solid was filtered off, and the solvent was evaporated under vacuum. The obtained residue was dissolved in ethyl acetate and the solution was extracted with brine. The organic phase was then dried with Na_2_SO_4_, filtered, and evaporated under vacuum, furnishing an oily residue. This residue was purified by chromatography on a silica gel column using a mixture of DCM/MeOH (9.5/0.5; *v*/*v*) as an eluent, thus obtaining the final product **2a** as an oil. Yield: 67%. ^1^H-NMR δ = 9.77 (s, 1H, -NH), 7.54 (d, *J =* 8.1 Hz, 2H), 7.19 (d, *J =* 8.1 Hz, 2H), 3.76–3.71 (m, 1H), 3.66–3.63 (m, 1H), 3.27–3.26 (m, 1H), 3.17–3.15 (m, 1H), 2.85–2.80 (m, 1H), 2.70–2.66 (m, 1H), 2.40–2.36 (m, 1H), 2.22–2.17 (m, 1H), 1.98–1.96 (m, 1H), 1.79–1.73 (m, 2H); ^13^C-NMR δ = 173.45, 136.99, 131.47, 129.11, 120.84, 68.18, 61.07, 57.89, 54.32, 30.87, 24.70. ESI-MS calculated: 268.74; Found: 269.3 [M + H]^+^. Anal. (C_13_H_17_ClN_2_O_2_), C, H, N.

#### 3.2.3. *N*-(4-hexylphenyl)-1-(2-hydroxyethyl)pyrrolidine-2-carboxamide (**2b**)

Compound **2b** was obtained following the procedure described for **2a**, starting from **1b** and 2-bromoethanol. Yield: 60%; oil. ^1^H-NMR δ = 9.65 (s, 1H, -NH), 7.53 (d, *J =* 8.1 Hz, 2H), 7.11 (d, *J* = 8.1 Hz, 2H), 3.78–3.75 (m, 1H), 3.72–3.70 (m, 1H), 3.33–3.31 (m, 1H), 3.24–3.20 (m, 1H), 2.88 (m, 1H), 2.74–2.71 (m, 1H), 2.56 (t, *J* = 7.0 Hz, *J* = 7.7 Hz, 2H), 2.46–2.43 (m, 1H), 2.20–2.25 (m, 1H), 2.01–2.05 (m, 1H), 1.82–1.80 (m, 2H), 1.28 (bs, 8H), 0.87 (t, *J* = 7.0 Hz, 3H); ^13^C-NMR δ = 173.24, 138.85, 135.88, 129.02, 119.54, 68.21, 61.09, 57.93, 54.36, 35.62, 31.97, 31.83, 30.88, 29.14, 24.65, 22.87, 14.38. ESI-MS calculated: 318.45; Found: 319.3 [M + H]^+^. Anal. (C_19_H_30_N_2_O_2_), C, H, N.

#### 3.2.4. *N*-(4-heptylphenyl)-1-(2-hydroxyethyl)pyrrolidine-2-carboxamide (**2c**)

Compound **2c** was obtained following the procedure described for **2a**, starting from **1c** and 2-bromoethanol. Yield: 54%; oil. ^1^H-NMR δ = 9.62 (s, 1H, -NH), 7.56 (d, *J* = 7.8 Hz, 2H), 7.11 (d, *J* = 7.8 Hz, 2H), 3.86–3.76 (m, 3H), 3.34–3.28 (m, 1H), 3.21–3.13 (m, 1H), 2.58 (t, *J* = 7.0 Hz, 2H), 2.45–2.39 (m, 1H), 2.09–2.01 (m, 1H), 2.03–1.97 (m, 1H), 1.78–1.73 (m, 2H), 1.69–1.65 (m, 1H), 1.52–1.45 (m, 2H), 1.30 (bs, 8H), 0.87 (t, *J* = 7.0 Hz, 3H); ^13^C-NMR δ = 172.65, 138.85, 135.41, 129.07, 119.50, 68.24, 61.10, 58.01, 54.37, 35.36, 31.99, 31.77, 30.87, 29.68, 29.14, 24.64, 22.64, 14.07. ESI-MS calculated: 332.48; Found: 333.4 [M + H]^+^. Anal. (C_20_H_32_N_2_O_2_), C, H, N.

#### 3.2.5. *N*-(4-octylphenyl)-1-(2-hydroxyethyl)pyrrolidine-2-carboxamide (**2d**)

Compound **2d** was obtained following the procedure described for **2a**, starting from **1d** and 2-bromoethanol. Yield: 73%; oil. ^1^H-NMR δ = 9.62 (s, 1H, -NH), 7.53 (d, *J* = 8.1 Hz, 2H), 7.10 (d, *J* = 8.1 Hz, 2H), 3.80–3.75 (m, 1H), 3.71–3.70 (m, 1H), 3.32–3.30 (m, 1H), 3.23–3.20 (m, 1H), 2.88 (m, 1H), 2.74–2.71 (m, 1H), 2.55 (t, *J* = 7.3 Hz, 2H), 2.47–2.41 (m, 1H), 2.24–2.22 (m, 1H), 2.03–2.00 (m, 1H), 1.82–1.80 (m, 2H), 1.49–1.52 (m, 2H), 1.26 (bs, 10H), 0.86 (t, *J* = 7.0 Hz, 3H); ^13^C-NMR δ = 173.04, 138.81, 135.90, 128.99, 119.52, 68.29, 61.13, 57.95, 54.38, 35.60, 32.10, 31.81, 30.87, 29.69, 29.49, 29.45, 24.63, 22.88, 14.32. ESI-MS calculated: 332.48; Found: 333.4 [M + H]^+^. Anal. (C_21_H_34_N_2_O_2_), C, H, N.

#### 3.2.6. 1-(2-hydroxyethyl)-*N*-(naphthalen-1-yl)pyrrolidine-2-carboxamide (**2e**)

Compound **2e** was obtained following the procedure described for **2a**, starting from **1e** and 2-bromoethanol. Yield: 66%; oil. ^1^H-NMR δ = 10.23 (s, 1H, –NH), 8.23 (d, *J* = 7.3 Hz, 1H), 8.01 (d, *J* = 7.7 Hz, 1H), 7.86 (d, 7.7 Hz, 1H), 7.65 (d, *J* = 8.1 Hz, 1H), 7.53–7.45 (m, 3H), 3.87–3.77 (m, 2H), 3.45–3.44 (m, 1H), 3.39 (dd, *J* = 13.9, 4.0 Hz, 1H), 3.05–3.00 (m, 1H), 2.82–2.77 (m, 1H), 2.56–2.50 (m, 1H), 2.36–2.26 (m, 1H), 2.13–2.10 (m, 1H), 1.93–1.90 (m, 2H); ^13^C-NMR δ = 173.65, 134.30, 132.90, 128.90, 126.54, 126.29, 126.16, 126.04,124.95, 120.91, 118.56, 68.90, 61.29, 58.26, 54.62, 31.09, 24.87. ESI-MS calculated: 284.15; Found: 285.3 [M + H]^+^. Anal. (C_17_H_20_N_2_O_2_), C, H, N.

#### 3.2.7. *N*-(biphenyl-2-yl)-1-(2-hydroxyethyl)pyrrolidine-2-carboxamide (**2f**)

Compound **2f** was obtained following the procedure described for **2a**, starting from **1f** and 2-bromoethanol. Yield: 72%; oil. ^1^H-NMR δ = 9.67 (s, 1H,–NH), 8.45 (d, *J* = 8.1 Hz, 1H), 7.50 (d, *J* = 7.3 Hz, 2H), 7.45–7.24 (m, 5H), 7.18 (t, *J* = 6.9 Hz, 1H), 3.28–3.25 (m, 3H), 3.19 (d, *J* = 9.9 Hz, 1H), 2.97–2.93 (m, 2H), 2.68–2.65 (m, 1H), 2.48–2.45 (m, 1H), 2.31–2.29 (m, 1H), 2.16–2.14 (m, 1H), 2.09–2.00 (m, 1H), 1.65–1.63 (m, 1H); ^13^C-NMR δ = 173.14, 139.08, 135.17, 132.25, 130.17, 129.80, 128.98, 128.80, 127.95, 124.23, 120.60, 68.88, 61.14, 58.16, 54.27, 30.85, 24.95. ESI-MS calculated: 310.39; Found: 311.4 [M + H]^+^. Anal. (C_19_H_22_N_2_O_2_), C, H, N.

#### 3.2.8. *N*-(biphenyl-4-yl)-1-(2-hydroxyethyl)pyrrolidine-2-carboxamide (**2g**)

Compound **2g** was obtained following the procedure described for **2a**, starting from **1g** and 2-bromoethanol. Yield: 69%; oil. ^1^H-NMR δ = 9.83 (s, 1H, –NH), 7.73 (d, *J* = 8.4 Hz, 2H), 7.57–7.53 (m, 4H), 7.43 (t, *J* = 7.3, 7.7 Hz, 2H), 7.33 (t, *J* = 7.3 Hz, 1H), 3.81–3.78 (m, 1H), 3.75–3.73 (m, 1H), 3.38–3.36 (m, 1H), 3.29–3.28 (m, 1H), 2.92–2.90 (m, 1H), 2.80–2.78 (m, 1H), 2.49–2.47 (m, 1H), 2.30–2.26 (m, 1H), 2.07–2.05 (m, 1H), 1.86–1.82 (m, 2H); ^13^C-NMR δ = 173.36, 140.92, 137.63, 136.95, 128.97, 128.80, 127.76, 127.04, 119.88, 68.28, 61.09, 57.93, 54.38, 30.89, 24.67. ESI-MS calculated: 310.39; Found: 311.3 [M + H]^+^. Anal. (C_19_H_22_N_2_O_2_), C, H, N.

#### 3.2.9. *N*-(4-chlorophenyl)-1-(3-hydroxypropyl)pyrrolidine-2-carboxamide (**3a**)

Compound **3a** was obtained following the procedure described for **2a**, starting from **1a** and 3-bromopropanol. Yield: 82%; oil. ^1^H-NMR δ = 9.47 (s, 1H, –NH), 7.60 (d, *J* = 8.1 Hz, 2H), 7.27 (d, *J* = 7.7 Hz, 2H), 3.83–3.76 (m, 2H), 3.30–3.27 (m, 1H), 3.17–3.14 (m, 1H), 2.93–2.86 (m, 1H), 2.61–2.58 (m, 1H), 2.40–2.33 (m, 1H), 2.30–2.20 (m, 1H), 1.98–1.96 (m, 1H), 1.83–1.78 (m, 4H); ^13^C-NMR δ = 173.20, 136.83, 131.47, 129.13, 120.86, 68.92, 61.39, 53.95, 53.14, 31.26, 30.71, 24.36. ESI-MS calculated: 282.77; Found: 283.3 [M + H]^+^. Anal. (C_14_H_19_ClN_2_O_2_), C, H, N.

#### 3.2.10. *N*-(4-hexylphenyl)-1-(3-hydroxypropyl)pyrrolidine-2-carboxamide (**3b**)

Compound **3b** was obtained following the procedure described for **2a**, starting from **1b** and 3-bromopropanol. Yield: 56%; oil. ^1^H-NMR δ = 9.65 (s, 1H, –NH), 7.53 (d, *J* = 8.1 Hz, 2H), 7.11 (d, *J* = 8.1 Hz, 2H), 3.78–3.75 (m, 2H), 3.72–3.70 (m, 1H), 3.33–3.31 (m, 1H), 3.24–3.20 (m, 1H), 2.88 (t, 2H), 2.74–2.71 (m, 1H), 2.56 (t, 2H), 2.46–2.43 (m, 1H), 2.20–2.25 (m, 1H), 2.01–2.05 (m, 1H), 1.82–1.80 (m, 1H), 1.28 (bs, 8H), 0.87 (t, 3H); ^13^C-NMR δ = 173.24, 138.98, 135.41, 128.89, 119.37, 68.21, 61.09, 53.89, 53.03, 35.35, 31.71, 31.51, 30.35, 29.68, 28.86, 23.93, 22.59, 14.08. ESI-MS calculated: 332.48; Found: 333.3 [M + H]^+^. Anal. (C_20_H_32_N_2_O_2_), C, H, N.

#### 3.2.11. *N*-(4-heptylphenyl)-1-(3-hydroxypropyl)pyrrolidine-2-carboxamide (**3c**)

Compound **3c** was obtained following the procedure described for **2a**, starting from **1c** and 3-bromopropanol. Yield: 84%; oil. ^1^H-NMR δ = 9.62 (s, 1H, –NH), 7.58 (d, *J* = 7.8 Hz, 2H), 7.12 (d, *J* = 7.8 Hz, 2H), 3.80–3.76 (m, 3H), 3.49–3.46 (m, 1H), 3.11–3.07 (m, 1H), 2.55 (t, *J* = 7.0 Hz, 2H), 2.47–2.40 (m, 1H), 2.14–2.09 (m, 1H), 2.03–1.97 (m, 1H), 1.82–1.80 (m, 2H), 1.70–1.66 (m, 1H), 1.58–1.55 (m, 2H), 1.30 (bs, 10H), 0.87 (t, *J* = 7.0 Hz, 3H); ^13^C-NMR δ = 172.80, 138.48, 135.41, 129.07, 119.50, 68.24, 61.08, 53.80, 53.13, 35.36, 31.50, 30.52, 29.67, 29.45, 29.24, 29.19, 24.14, 22.68, 14.07. ESI-MS calculated: 346.51; Found: 347.4 [M + H]^+^. Anal. (C_21_H_34_N_2_O_2_), C, H, N.

#### 3.2.12. *N*-(4-octylphenyl)-1-(3-hydroxypropyl)pyrrolidine-2-carboxamide (**3d**)

Compound **3d** was obtained following the procedure described for **2a**, starting from **1d** and 3-bromopropanol. Yield: 61%; oil. ^1^H-NMR δ = 9.62 (s, 1H, –NH), 7.50 (d, *J* = 7.8 Hz, 2H), 7.13 (d, *J* = 7.8 Hz, 2H), 3.90–3.85 (m, 2H), 3.76–3.72 (m, 1H), 3.64–3.62 (m, 1H), 3.50–3.49 (m, 1H), 3.26–3.24 (m, 1H), 3.12–3.10 (m, 1H), 3.02–2.99 (m, 1H), 2.53 (t, *J* = 6.6 Hz, 2H), 2.03–2.00 (m, 1H), 1.92–1.94 (m, 2H), 1.76–1.78 (m, 2H), 1.49–1.52 (m, 2H), 1.26 (bs, 10H), 0.86 (t, *J* = 7.0 Hz, 3H); ^13^C-NMR δ = 172.93, 138.99, 135.48, 128.87, 119.45, 68.54, 60.82, 53.80, 52.88, 35.35, 31.86, 31.55, 30.52, 29.67, 29.45, 29.24, 29.19, 24.15, 22.64, 14.08. ESI-MS calculated: 360.53; Found: 361.6 [M + H]^+^. Anal. (C_22_H_36_N_2_O_2_), C, H, N.

#### 3.2.13. 1-(3-hydroxypropyl)-*N*-(naphthalen-1-yl)pyrrolidine-2-carboxamide (**3e**)

Compound **3e** was obtained following the procedure described for **2a**, starting from **1e** and 3-bromopropanol. Yield: 52%; oil. ^1^H-NMR δ = 10.11 (s, 1H, –NH), 8.20 (d, *J* = 7.3 Hz, 1H), 7.87–7.84 (m, 2H), 7.66 (d, *J* = 8.1 Hz, 1H), 7.55–7.46 (m, 3H), 3.77–3.74 (m, 2H), 3.42–3.40 (m, 1H), 3.35 (dd, *J* = 13.9, 4.0 Hz, 1H), 3.02–2.96 (m, 1H), 2.76–2.70 (m, 1H), 2.52–2.46 (m, 1H), 2.34–2.29 (m, 1H), 2.11–2.08 (m, 1H), 1.91–1.88 (m, 4H); ^13^C-NMR δ = 173.52, 134.32, 132.72, 129.08, 126.54, 126.38, 126.23, 126.07, 125.01, 120.42, 118.76, 69.09, 61.24, 54.44, 53.40, 32.00, 31.02, 24.79. ESI-MS calculated: 298.29; Found: 299.1 [M + H]^+^. Anal. (C_18_H_22_N_2_O_2_), C, H, N.

#### 3.2.14. *N*-(biphenyl-2-yl)-1-(3-hydroxypropyl)pyrrolidine-2-carboxamide (**3f**)

Compound **3f** was obtained following the procedure described for **2a**, starting from **1f** and 3-bromopropanol. Yield: 62%; oil. ^1^H-NMR δ = 9.71 (s, 1H, –NH), 8.37 (d, *J* = 8.1 Hz, 1H), 7.46 (d, *J* = 6.6 Hz, 2H), 7.39–7.23 (m, 5H), 7.18 (t, *J* = 6.9 Hz, 1H), 3.32–3.16 (m, 3H), 2.96–2.91 (m, 2H), 2.58–2.55 (m, 1H), 2.43–2.40 (m, 1H), 2.28–2.24 (m, 1H), 2.16–2.14 (m, 1H), 2.02–1.94 (m, 1H), 1.78–1.75 (m, 2H), 1.63–1.60 (m, 1H); ^13^C-NMR δ = 173.14, 139.10, 135.17, 132.25, 130.16, 129.63, 128.98, 128.80, 127.95, 124.33, 120.61, 68.90, 61.15, 54.24, 53.38, 31.25, 30.85, 24.95. ESI-MS calculated: 324.42; Found: 325.4 [M + H]^+^. Anal. (C_20_H_24_N_2_O_2_), C, H, N.

#### 3.2.15. *N*-(biphenyl-4-yl)-1-(3-hydroxypropyl)pyrrolidine-2-carboxamide (**3g**)

Compound **3g** was obtained following the procedure described for **2a**, starting from **1g** and 3-bromopropanol. Yield: 54%; oil. ^1^H-NMR δ = 9.76 (s, 1H, –NH), 7.74 (d, *J* = 8.2 Hz, 2H), 7.55–7.53 (m, 4H), 7.42 (t, *J* = 7.4, 7.0 Hz, 2H), 7.33 (t, *J* = 7.4 Hz, 1H), 3.82–3.78 (m, 2H), 3.65–3.63 (m, 1H), 3.48–3.42 (m, 1H), 3.06–3.00 (m, 1H), 2.87–2.84 (m, 2H), 2.62–2.60 (m, 1H), 2.40–2.37 (m, 1H), 2.05–2.03 (m, 1H), 1.90–1.83 (m, 3H); ^13^C-NMR δ = 173.36, 140.48, 137.13, 137.05, 128.77, 127.55, 127.09, 126.80, 119.99, 68.54, 61.02, 54.04, 53.37, 30.45, 29.68, 23.91. ESI-MS calculated: 324.42; Found: 325.5 [M + H]^+^. Anal. (C_20_H_24_N_2_O_2_), C, H, N.

### 3.3. Synthesis of 1,4-Disubstituted 1,2,3-Triazoles **5a**–**5h**

#### 3.3.1. Synthesis of 1-Azido-4-octylbenzene (**4**)

One mmol of 4-octylaniline was suspended in 1 mL of H_2_O and 0.370 mL of concentrated HCl. To the reaction mixture, stirred and cooled to 0 °C, a solution of NaNO_2_ (1.04 mmol) in 1 mL of H_2_O was then added dropwise. After 30 min, the reaction mixture containing the formed diazonium salt, was poured into a solution of sodium azide (1.12 mmol) in 0.2 mL of H_2_O and ice. The formation of an intense foam, due to the release of N_2_, was observed. The mixture was stirred for 12 h, then the solution was extracted with DCM (three times) and the collected organic phases were dried with Na_2_SO_4_. Evaporation of the solvent left the desired intermediate **4**, with a yield of 68%.

#### 3.3.2. Synthesis of (1-(4-Octylphenyl)-1H-1,2,3-triazol-4-yl)methanol (**5a**)

2-Propin-1-ol (1 mmol) and **4** (1 mmol) were suspended in a 1:1 mixture of water and tert-butyl alcohol (12 mL). Sodium ascorbate (0.05 mmol) and copper sulfate pentahydrate (0.01 mmol) dissolved in 6 mL of H_2_O:tBuOH (1/1, *v*/*v*) were added. The heterogeneous mixture was stirred vigorously for 48 h, at which point it cleared and TLC analysis indicated complete consumption of the reactants. The reaction mixture was diluted with water (15 mL), cooled in ice, and the white precipitate was collected by filtration. After washing the precipitate with cold water (2 × 5 mL), it was dried under vacuum to afford the pure product as an off-white powder. Yield: 85%; mp 69–70 °C. ^1^H-NMR δ = 8.59 (s, 1H), 7.76 (d, *J* = 7.0 Hz, 2H), 7.37 (d, *J* = 6.6 Hz, 2H), 5.27 (s, 2H), 4.58 (s, 1H, –OH), 2.61 (m, *J* = 7.8 Hz, *J* = 6.3 Hz, 2H), 1.57 (m, 2H), 1.26 (bs, 10 H), 0.82 (t, *J* = 6.3 Hz, *J* = 7.0 Hz, 3H). ^13^C-NMR δ = 149.66, 143.59, 135.36, 130.24, 121.55, 120.56, 55.65, 35.24, 31.94, 31.48, 29.49, 29.33, 29.29, 22.75, 14.63. ESI-MS calculated: 287.40; Found: 288.0 [M + H]^+^. Anal. (C_17_H_25_N_3_O), C, H, N.

#### 3.3.3. 2-(1-(4-Octylphenyl)-1H-1,2,3-triazol-4-yl)ethanol (**5b**)

Compound **5b** was obtained following the procedure described for **5a**, starting from **4** and 3-butyn-1-ol. Yield: 90%; mp 90–91 °C. ^1^H-NMR δ = 8.48 (s, 1H), 7.74 (d, *J* = 7.7 Hz, 2H), 7.37 (d, *J* = 7.7 Hz, 2H), 4.72 (s, 1H, –OH), 3.68 (m, 2H), 2.84 (t, *J* = 6.2 Hz, *J* = 6.6 Hz, 2H), 2.61 (t, *J* = 6.9 Hz, 2H), 1.58 (m, 2H), 1.27 (bs, 10 H), 0.83 (t, *J* = 6.3 Hz, *J* = 7.0 Hz, 3H). ^13^C-NMR δ = 146.17, 143.44, 135.38, 130.23, 121.30, 120.44, 60.93, 35.24, 31.95, 31.47, 29.88, 29.49, 29.33, 29.28, 22.76, 14.63. ESI-MS calculated: 301.43; Found: 302.1 [M + H]^+^. Anal. (C_18_H_27_N_3_O) C, H, N.

#### 3.3.4. 3-(1-(4-Octylphenyl)-1H-1,2,3-triazol-4-yl)propan-1-ol (**5c**)

Compound **5c** was obtained following the procedure described for **5a**, starting from **4** and 4-pentyn-1-ol. Yield: 77%; mp 59–60 °C. ^1^H-NMR δ = 8.48 (s, 1H), 7.74 (d, *J* = 8.2 Hz, 2H), 7.37 (d, *J* = 7.7 Hz, 2H), 4.50 (t, 1H, –OH, *J* = 5.1 Hz), 3.48 (m, 2H), 2.73 (t, 2H, *J* = 7.4 Hz, *J* = 7.8 Hz), 2.63 (t, 2H, *J* = 7.4 Hz, *J* = 7.8 Hz), 1.82 (m, 2H), 1.57 (m, 2H), 1.27 (bs, 10 H), 0.85 (t, 3H, *J* = 6.3 Hz, *J* = 6.6 Hz). ^13^C-NMR δ = 148.35, 143.17, 135.20, 129.97, 120.43, 120.18, 60.46, 34.99, 32.58, 31.70, 31.25, 29.25, 29.09, 29.04, 22.52, 22.14, 14.40. ESI-MS calculated: 315.45; Found: 316.1 [M + H]^+^; 338.3 [M + Na]^+^; 354.3 [M + K]^+^. Anal. (C_19_H_29_N_3_O), C, H, N.

#### 3.3.5. 2-(1-(4-Octylphenyl)-1H-1,2,3-triazol-4-yl)pyridine (**5d**)

Compound **5d** was obtained following the procedure described for **5a**, starting from **4** and 2-ethynylpyridine. Yield: 83%; mp 85–86 °C. ^1^H-NMR δ = 9.25 (s, 1H), 8.64 (d, *J* = 3.9 Hz, 1H), 8.10 (d, *J* = 7.8 Hz, 1H), 7.92 (m, 3H), 7.41 (m, 3H), 2.65 (t, *J* = 7.4 Hz, *J* = 7.8 Hz, 2H), 1.58 (m, 2H), 1.28 (bs, 10 H), 0.83 (t, *J* = 6.3 Hz, *J* = 6.6 Hz, 3H). ^13^C-NMR δ = 150.10, 150.03, 148.54, 143.78, 137.77, 134.93, 130.05, 123.74, 121.58, 120.57, 120.21, 35.03, 31.71, 31.24, 29.26, 29.10, 29.06, 22.52, 14.39. ESI-MS calculated: 334.46; Found: 335.2 [M + H]^+^. Anal. (C_21_H_26_N_4_), C, H, N.

#### 3.3.6. 3-(1-(4-Octylphenyl)-1H-1,2,3-triazol-4-yl)pyridine (**5e**)

Compound **5e** was obtained following the procedure described for **5a**, starting from **4** and 3-ethynylpyridine. Yield: 85%; mp 125–126 °C. ^1^H-NMR δ = 9.35 (s, 1H), 9.12 (s, 1H), 8.57 (m, 2H), 8.29 (d, *J* = 7.7 Hz, 1H), 7.81 (d, *J* = 8.1 Hz, 2H), 7.44 (d, *J* = 8.1 Hz, 2H), 2.64 (t, *J* = 7.4 Hz, *J* = 7.8 Hz, 2H), 1.59 (m, 2H), 1.27 (bs, 10 H), 0.83 (t, *J* = 6.3 Hz, *J* = 6.6 Hz, 3H). ^13^C-NMR δ = 149.7, 146.97, 144.87, 143.88, 134.90, 132.97, 130.13, 124.54, 120.78, 120.50, 79.62, 35.05, 31.71, 31.25, 29.26, 29.11, 22.53, 14.40. ESI-MS calculated: 334.46; Found: 335.2 [M + H]^+^. Anal. (C_21_H_26_N_4_), C, H, N.

#### 3.3.7. 1-(1-(4-Octylphenyl)-1H-1,2,3-triazol-4-yl)cyclohexanol (**5f**)

Compound **5f** was obtained following the procedure described for **5a**, starting from **4** and 1-ethynylcyclohexanol. Yield: 78%; mp 119–120 °C. ^1^H-NMR δ = 8.51 (s, 1H), 7.77 (d, *J* = 8.1 Hz, 2H), 7.36 (d, *J* = 8.1 Hz, 2H), 4.94 (s, 1H), 2.62 (t, *J* = 7.0 Hz, *J* = 7.7 Hz, 2H), 1.94–1.63 (mm, 6H), 1.57 (m, 2H), 1.43 (m, 4H), 1.26 (bs, 10 H), 0.82 (t, *J* = 6.3 Hz, *J* = 6.6 Hz, 3H). ^13^C-NMR δ = 157.35, 143.41, 135.46, 130.21, 120.44, 119.85, 68.64, 38.37, 35.24, 31.95, 31.47, 29.49, 29.30, 25.93, 22.76, 22.38, 14.63. ESI-MS calculated: 355.52; Found: 356.4 [M + H]^+^. Anal. (C_22_H_33_N_3_O), C, H, N.

#### 3.3.8. (4-(1-(4-Octylphenyl)-1H-1,2,3-triazol-4-yl)phenyl)methanol (**5g**)

Compound **5g** was obtained following the procedure described for **5a**, starting from **4** and (4-ethynylphenyl)methanol. Yield: 87%; mp 160–161 °C. ^1^H-NMR δ = 9.20 (s, 1H), 7.89 (d, *J* = 7.0 Hz, 2H), 7.83 (d, *J* = 7.4 Hz, 2H), 7.43 (d, *J* = 7.4 Hz, 4H) 5.23 (s, 1H, -OH), 4.53 (d, *J* = 4.7 Hz, 2H), 2.66 (t, *J* = 7.0 Hz, *J* = 6.6 Hz, 2H), 1.60 (m, 2H), 1.28 (bs, 10 H), 0.84 (t, *J* = 6.3 Hz, *J* = 6.6 Hz, 3H). ^13^C-NMR δ = 147.67, 143.60, 143.07, 135.03, 130.06, 129.18, 127.41, 125.52, 120.34, 119.70, 63.10, 35.03, 31.70, 31.24, 29.25, 29.10, 29.06, 22.51, 14.39. ESI-MS calculated: 363.50; Found: 364.1 [M + H]^+^. Anal. (C_23_H_29_N_3_O), C, H, N.

#### 3.3.9. 3-(1-(4-Octylphenyl)-1H-1,2,3-triazol-4-yl)phenol (**5h**)

Compound **5h** was obtained following the procedure described for **5a**, starting from **4** and 2-ethynylphenol. Yield: 78%; mp 126–127 °C. ^1^H-NMR δ = 9.57 (s, 1H, –OH), 9.15 (s, 1H), 7.82 (d, *J* = 7.3 Hz, 2H), 7.41 (d, *J* = 7.4 Hz, 2H), 7.35 (s, 1H), 7.32 (d, *J* = 6.6 Hz, 1H), 7.27 (t, *J* = 7.9 Hz, *J* = 6.3 Hz, *J* = 7.0 Hz, 1H), 6.75 (d, *J* = 7.4 Hz, 1H), 2.63 (t, *J* = 7.0 Hz, *J* = 6.6 Hz, 2H), 1.58 (m, 2H), 1.27 (bs, 10 H), 0.83 (t, *J* = 6.3 Hz, *J* = 6.6 Hz, 3H). ^13^C-NMR δ = 158.27, 147.75, 143.59, 135.03, 131.96, 130.45, 130.05, 120.37, 119.86, 116.67, 115.67, 112.53, 35.03, 31.71, 31.25, 29.26, 29.10, 29.05, 22.52, 14.39. ESI-MS calculated: 349.42; Found: 350.2 [M + H]^+^. Anal. (C_22_H_27_N_3_O), C, H, N.

### 3.4. Synthesis of 1,4-Disubstituted 1,2,3-Triazoles **8a**–**8h**

#### 3.4.1. Synthesis of 2-Bromo-*N*-(4-octylphenyl)acetamide (**6**)

The 2-bromoacetyl bromide (1.3 mmol), dissolved in toluene (5 mL), was added dropwise to a solution of 4-octyl-aniline (1 mmol) in toluene (5 mL). The reaction mixture was heated to 110 °C, and after 4 h, ethyl acetate was added and the resulting solution was extracted with brine (three times). The organic phase, dried with Na_2_SO_4_, was filtered and evaporated to dryness. The crystallization with ethyl ether provided the desired product **6** (yield: 83%).

#### 3.4.2. Synthesis of 2-Azido-*N*-(4-octylphenyl)acetamide (**7**)

The intermediate **6** (1 mmol) was dissolved in ethanol (10 mL), sodium azide (1.25 mmol) was added, and the reaction was refluxed for 2 h and 30 min. Then, it was cooled and diethyl ether (10 mL) was added. The precipitated NaBr was filtered off and the filtrate was evaporated to dryness. The residue was crystallized with hexane providing the desired product with **7** (yield: 86%).

#### 3.4.3. 2-(4-(Hydroxymethyl)-1H-1,2,3-triazol-1-yl)-*N*-(4-octylphenyl)acetamide (**8a**)

Compound **8a** was obtained following the procedure described for **5a**, starting from **7** and 2-propin-1-ol. Yield: 75%; mp 171–172 °C. ^1^H-NMR δ = 10.34 (s, 1H, –NH), 7.95 (s, 1H), 7.46 (d, *J* = 6.2 Hz, 2H), 7.12 (d, *J* = 6.3 Hz, 2H), 5.26 (s, 2H), 5.18 (s, 1H, –OH), 4.52 (s, 2H), 2.48 (t, 2H, *J* = 7.8 Hz, *J* = 6.3 Hz), 1.51 (m, 2H), 1.24 (bs, 10 H), 0.83 (t, 3H, *J* = 6.3 Hz, *J* = 7.0 Hz). ^13^C-NMR δ = 169.46, 153.23, 143.19, 141.54, 134.03, 129.76, 124.66, 60.47, 57.52, 39.97, 36.69, 36.40, 34.25, 34.09, 34.03, 27.51, 19.38. ESI-MS calculated: 344.45; Found: 345.5 [M + H]^+^. Anal. (C_19_H_28_N_4_O_2_), C, H, N.

#### 3.4.4. 2-(4-(2-Hydroxyethyl)-1H-1,2,3-triazol-1-yl)-*N*-(4-octylphenyl)acetamide (**8b**)

Compound **8b** was obtained following the procedure described for **5a**, starting from **7** and 3-butyn-1-ol. Yield: 80%; mp 170–171 °C. ^1^H-NMR δ = 10.32 (s, 1H, –NH), 7.85 (s, 1H), 7.45 (d, *J* = 7.0 Hz, 2H), 7.12 (d, *J* = 7.0 Hz, 2H), 5.21 (s, 2H), 4.67 (s, 1H, –OH), 3.63 (m, 2H), 2.77 (t, 2H, *J* = 6.6 Hz, *J* = 6.2 Hz), 2.49 (t, 2H, *J* = 7.8 Hz, *J* = 6.3 Hz), 1.51 (m, 2H), 1.23 (bs, 10 H), 0.82 (t, 3H, *J* = 6.3 Hz, *J* = 7.0 Hz). ^13^C-NMR δ = 164.50, 144.64, 138.19, 136.54, 129.04, 124.46, 119.67, 60.84, 52.51, 34.98, 31.70, 31.40, 29.59, 29.26, 29.10, 29.04, 22.51, 14.39. ESI-MS calculated: 358.48; Found: 359.3 [M + H]^+^. Anal. (C_20_H_30_N_4_O_2_), C, H, N.

#### 3.4.5. 2-(4-(3-Hydroxypropyl)-1H-1,2,3-triazol-1-yl)-*N*-(4-octylphenyl)acetamide (**8c**)

Compound **8c** was obtained following the procedure described for **5a**, starting from **7** and 4-pentyn-1-ol. Yield: 82%; mp 171–172 °C. ^1^H-NMR δ = 10.30 (s, 1H, –NH), 7.81 (s, 1H), 7.43 (d, *J* = 7.0 Hz, 2H), 7.10 (d, *J* = 7.0 Hz, 2H), 5.19 (s, 2H), 4.44 (s, 1H, –OH), 3.40 (m, 2H), 2.62 (t, 2H, *J* = 7.4 Hz, *J* = 7.8 Hz), 1.71 (t, 2H, *J* = 7.4 Hz, *J* = 7.8 Hz), 1.49 (m, 2H), 1.21 (bs, 10 H), 0.81 (t, 3H, *J* = 6.3 Hz, *J* = 7.0 Hz). ^13^C-NMR δ = 164.53, 146.99, 138.19, 136.54, 129.03, 123.88, 119.67, 60.49, 52.50, 34.98, 32.74, 31.70, 31.40, 29.26, 29.10, 29.04, 22.51, 22.07, 14.39. ESI-MS calculated: 372.50; Found: 373.3 [M + H]^+^. Anal. (C_21_H_32_N_4_O_2_), C, H, N.

#### 3.4.6. *N*-(4-octylphenyl)-2-(4-(pyridin-2-yl)-1H-1,2,3-triazol-1-yl)acetamide (**8d**)

Compound **8d** was obtained following the procedure described for **5a**, starting from **7** and 2-ethynylpyridine. Yield: 78%; mp 199–200 °C. ^1^H-NMR δ = 10.40 (s, 1H, –NH), 8.59 (s, 1H), 8.04 (d, *J* = 7.7 Hz, 1H), 7.91 (m, 2H), 7.47 (d, *J* = 8.1 Hz, 2H), 7.35 (t, 1H, *J* = 5.5 Hz, *J* = 6.2 Hz), 7.13 (d, *J* = 8.1 Hz, 2H), 5.38 (s, 2H), 2.51 (t, 2H, *J* = 7.8 Hz, *J* = 6.3 Hz), 1.51 (m, 2H), 1.24 (bs, 10 H), 0.84 (t, 3H, *J* = 6.3 Hz, *J* = 7.0 Hz). ^13^C-NMR δ = 164.53, 150.68, 150.35, 147.74, 138.49, 137.89, 136.76, 129.30, 125.65, 123.63, 120.08, 119.96, 52.99, 35.23, 31.94, 31.64, 29.50, 29.34, 29.28, 22.75, 14.63. ESI-MS calculated: 391.51; Found: 392.2 [M + H]^+^. Anal. (C_23_H_29_N_5_O), C, H, N.

#### 3.4.7. *N*-(4-octylphenyl)-2-(4-(pyridin-3-yl)-1H-1,2,3-triazol-1-yl)acetamide (**8e**)

Compound **8e** was obtained following the procedure described for **5a**, starting from **7** and 3-ethynylpyridine. Yield: 85%; mp 196–197 °C. ^1^H-NMR δ = 10.42 (s, 1H, –NH), 9.06 (s, 1H), 8.71 (s, 1H), 8.53 (m, 2H), 8.23 (d, *J* = 7.7 Hz, 1H), 7.47 (d, *J* = 8.1 Hz, 2H), 7.13 (d, *J* = 8.1 Hz, 2H), 5.37 (s, 2H), 2.51 (t, 2H, *J* = 7.8 Hz, *J* = 6.3 Hz), 1.51 (m, 2H), 1.23 (bs, 10 H), 0.84 (t, 3H, *J* = 6.3 Hz, *J* = 7.0 Hz). ^13^C-NMR δ = 164.48, 149.57, 147.04, 144.10, 138.55, 136.72, 133.09, 129.31, 124.72, 124.42, 119.97, 94.57, 53.11, 35.21, 31.93, 31.63, 29.49, 29.33, 29.27, 22.75, 14.63. ESI-MS calculated: 391.51; Found: 392.1 [M + H]^+^. Anal. (C_23_H_29_N_5_O), C, H, N.

#### 3.4.8. 2-(4-(1-Hydroxycyclohexyl)-1H-1,2,3-triazol-1-yl)-*N*-(4-octylphenyl)acetamide (**8f**)

Compound **8f** was obtained following the procedure described for **5a**, starting from **7** and 1-ethynylcyclohexanol. Yield: 83%; mp 154–155 °C. ^1^H-NMR δ = 10.33 (s, 1H, –NH), 7.86 (s, 1H), 7.46 (d, *J* = 8.2 Hz, 2H), 7.12 (d, *J* = 8.2 Hz, 2H), 5.23 (s, 2H), 4.86 (s, 1H, –OH), 2.51 (t, 2H, *J* = 7.8 Hz, *J* = 6.3 Hz), 1.85–1.64 (mm, 6H), 1.51 (m, 2H), 1.41 (m, 4H), 1.24 (bs, 10 H), 0.83 (t, 3H, *J* = 6.3 Hz, *J* = 7.0 Hz). ^13^C-NMR δ = 164.51, 156.08, 138.19, 136.57, 129.04, 122.97, 119.65, 68.44, 52.55, 38.29, 34.98, 31.70, 31.41, 29.26, 29.10, 29.04, 25.70, 22.52, 22.09, 14.39. ESI-MS calculated: 412.57; Found: 413.3 [M + H]^+^. Anal. (C_24_H_36_N_4_O_2_), C, H, N.

#### 3.4.9. 2-(4-(4-(Hydroxymethyl)phenyl)-1H-1,2,3-triazol-1-yl)-*N*-(4-octylphenyl)acetamide (**8g**)

Compound **8g** was obtained following the procedure described for **5a**, starting from **7** and (4-ethynylphenyl)methanol. Yield: 78%; mp 238–239 °C. ^1^H-NMR δ = 10.39 (s, 1H, –NH), 8.52 (s, 1H), 7.81 (d, *J* = 7.8 Hz, 2H), 7.47 (d, *J* = 8.6 Hz, 2H), 7.38 (d, *J* = 7.8 Hz, 2H), 7.12 (d, *J* = 8.6 Hz, 2H), 5.32 (s, 2H), 5.20 (t, 1H, –OH, *J* = 5.9 Hz, *J* = 5.9 Hz), 4.51 (d, *J* = 5.5 Hz, 2H) 2.51 (t, 2H, *J* = 7.8 Hz, *J* = 6.3 Hz), 1.50 (m, 2H), 1.23 (bs, 10 H), 0.82 (t, 3H, *J* = 6.3 Hz, *J* = 7.0 Hz). ^13^C-NMR δ = 164.36, 146.65, 142.68, 138.26, 136.51, 129.61, 129.06, 127.40, 125.34, 123.22, 119.71, 63.11, 52.778, 34.98, 31.69, 31.39, 29.25, 29.10, 29.03, 22.51, 14.38. ESI-MS calculated: 420.55; Found: 421.2 [M + H]^+^. Anal. (C_25_H_32_N_4_O_2_), C, H, N.

#### 3.4.10. 2-(4-(3-Hydroxyphenyl)-1H-1,2,3-triazol-1-yl)-*N*-(4-octylphenyl)acetamide (**8h**)

Compound **8h** was obtained following the procedure described for **5a**, starting from **7** and 2-ethynylphenol. Yield: 83%; mp 200–201 °C. ^1^H-NMR δ = 10.39 (s, 1H, –NH), 9.51 (s, 1H, –OH), 8.48 (s, 1H), 7.47 (d, *J* = 7.8 Hz, 2H), 7.27-7.21 (m, 3H), 7.13 (d, *J* = 8.2 Hz, 2H), 6.72 (s, 1H), 5.31 (s, 2H), 2.51 (t, 2H, *J* = 7.8 Hz, *J* = 6.3 Hz), 1.51 (m, 2H), 1.23 (bs, 10 H), 0.82 (t, 3H, *J* = 6.3 Hz, *J* = 7.0 Hz). ^13^C-NMR δ = 164.36, 158.22, 146.71, 138.26, 136.52, 132.38, 130.41, 129.06, 123.41, 119.72, 116.48, 115.34, 112.30, 52.75, 34.98, 31.71, 31.40, 29.26, 29.10, 29.04, 22.51, 14.39. ESI-MS calculated: 406.52; Found: 407.3 [M + H]^+^. Anal. (C_24_H_30_N_4_O_2_), C, H, N.

### 3.5. Biological Assays

#### 3.5.1. Aorta Rings Assay

Male CD-1 mice (18 ± 2 g body weight, Charles River, Calco, Italy) were housed in a controlled environment (21 ± 2 °C) and provided with standard rodent chow and water. All animals were allowed to acclimate for four days prior to experiments and were subjected to a 12 h light–12 h dark schedule. Experiments were conducted during the light phase. The experimental procedures, according to Italian (DL 26/2014) and European (No. 63/2010/UE) regulations on the protection of animals used for experimental and other scientific purposes, were approved by the Italian Ministry (No. 958/2015-PR, 14 September 2015). Mice were sacrificed to take the thoracic aorta. Aortas were cleaned of adherent connective tissue and cut into rings 2 mm in length. Aortic rings were mounted on 2.5 mL organ baths containing Krebs salt solution of the following composition (in mM): NaCl, 118.4; KCl, 4.7; MgSO_4_ 1.2; CaCl_2_, 1.3; KH_2_PO_4_, 1.2; NaHCO_3_ 25.0; and glucose 11.7. The solution was maintained at 37 °C and bubbled with 95% O_2_–5% CO_2_ (pH 7.4). Developed tension was measured using an isometric force transducer (Basile; Italy) connected to a recorder. Rings were initially stretched until a resting tension of 1g was reached and allowed to equilibrate for at least 30 min during which tension was adjusted, when necessary, to 1 g and the bathing solution was periodically changed. In each experiment, aortic rings were firstly challenged with PE (10^−6^ M) until the responses were reproducible. Subsequently, after tissue washing, a cumulative concentration response curve to acetylcholine (10^−8^ to 3 × 10^−5^ M) was performed.

#### 3.5.2. Compounds and Cell Cultures

Fingolimod and PF-543 were purchased from Selleck Chemicals (München, Germany). A549, H1975 and HCC827 human NSCLC cell lines were obtained from the American Type Culture Collection (ATCC). These cell lines express both SphK1 and SphK2 (data not shown). All cell lines were authenticated using Short Tandem Repeat (STR) DNA profiling and maintained in RPMI-1640 medium supplemented with 10% heat-inactivated fetal bovine serum, 20 mM 4-(2-hydroxyethyl)-1-piperazineethanesulfonic acid (HEPES), pH 7.4, penicillin (100 IU/mL), streptomycin (100 µg/mL), and 4 mM glutamine (ICN, Irvine, UK) in a humified atmosphere of 95% air and 5% CO_2_ at 37 °C.

#### 3.5.3. Cell Density Assay (3-(4,5-dimethylthiazol-2-yl)-2,5-diphenyltetrazolium bromide (MTT))

Cells (10^4^ cells/well) were grown in 24-well plates and exposed for 72 h to increasing doses of the compounds (0.5 to 5 µM). The percentage of cell density was determined by using the 3-(4,5-dimethylthiazol-2-yl)-2,5-diphenyltetrazolium bromide (MTT). The dose of each compound able to reduce cell density by 50% was used as a marker of drug effect.

#### 3.5.4. Sphingosine Kinase Activity Assay

The capability of the tested compounds to inhibit SphK1 and SphK2 enzyme activity was measured by using the Sphingosine Kinase Activity Assay (K-3500, Echelon Biosciences, Salt Lake City, UT, USA). The assay was performed as indicated by the manufacturer, by using sphingosine 100 µM and ATP 10 µM. SphK1 (E-K068, Echelon Biosciences) and SphK2 (E-K069, Echelon Biosciences) were added at a concentration of 500 ng/mL. PF-543 and the compounds **5h** and **8f** were tested at 10 µM.

#### 3.5.5. Statistical Analysis

The results of cell density assays were analyzed by Student’s t test and expressed as means and standard deviations (SDs) for three independent experiments performed in triplicates. The linear regression test was used to evaluate the statistical significance of the results of the Sphingosine Kinase activity assay (Graph-Pad version 5 (La Jolla, CA, USA). All reported *P* values were two-sided. Analyses were performed with the BMDP New System statistical package version 1.0 for Microsoft Windows (BMDP Statistical Software, Los Angeles, CA, USA).

## 4. Conclusions

The aim of the present study was to identify novel SphK1 and/or SphK2 inhibitors useful in further exploring the use of these pharmacological targets in the anticancer research field. According to the finding that a key characteristic feature for most of the already reported inhibitors is a highly lipophilic backbone connected by a linker to a polar (mainly basic) head group, we started this project with the synthesis of two series of compounds (**2a**–**2g** and **3a**–**3g**) where a pyrrolidine spacer was used to link several lipophilic tails to a two- or three-unit polar head. Only the 4-octylphenyl derivatives **2d** and **3d** showed an inhibitory activity in the aorta rings based assay and for this reason this scaffold was selected for further development of this project. Two series of 1,4-disubstituted 1,2,3-triazoles (**5a**–**5h** and **8a**–**8h**) were synthesized and evaluated for their ability to interfere with the Ach induced relaxation of aortic rings. Then, their capability in inhibiting proliferation of several cell lines expressing both SphK1 and SphK2 was investigated. Compounds **5h** and **8f** were the most interesting in terms of antiproliferative activity, and their selectivity towards the two isoforms was tested. Compound **8f** produced a more pronounced inhibition of SphK1 than SphK2, while **5h** inhibited both the enzyme isoforms. Co-crystal structures of SphK1 with three inhibitors have been obtained [17,23,24]. All the crystallized inhibitors occupy the J-shaped channel where the alkyl chain of S1P resides. Key residues in the binding site are largely conserved between SphK1 and SphK2 [25]. The molecular model of **5h** shows that it can adapt to both isoforms because of its simpler and shorter structure. Compound **8f** is not only able to accommodate the 4-octylphenyl moiety in the J channel, but it presents a higher complementarity with the active site of SphK1 due to its flexible and polar head; that makes this compound a novel selective inhibitor of SphK1.

## Figures and Tables

**Table 1:** Structures of the 1,4-disubstituted 1,2,3-triazoles **5a**–**h** and **8a**–**h**. 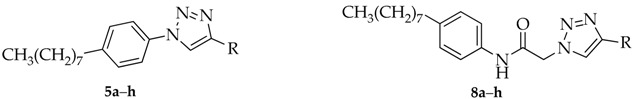

Code	R	% of Inhibition on Aorta Rings	Dose Able to Reduce Cell Density of 50%
**5a**	–CH_2_OH	67	>10 µM
**5b**	–(CH_2_)_2_OH	63	>10 µM
**5c**	–(CH_2_)_3_OH	64	5–10 µM
**5d**	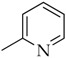	35	>10 µM
**5e**	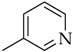	n.a. *	>10 µM
**5f**	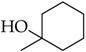	26	5–10 µM
**5g**	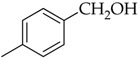	21	>10 µM
**5h**	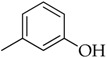	46	≤5 µM
**8a**	–CH_2_OH	65	>10 µM
**8b**	–(CH_2_)_2_OH	18	>10 µM
**8c**	–(CH_2_)_3_OH	19.5	>10 µM
**8d**	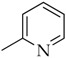	n.a. *	>10 µM
**8e**	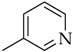	n.a. *	>10 µM
**8f**	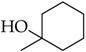	46	≤5 µM
**8g**	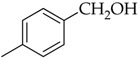	16	>10 µM
**8h**	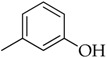	42	>10 µM

* n.a. = not active.

## Supplementary Materials

Supplementary materials can be found at www.mdpi.com/1422-0067/18/11/2332/s1.
