# Supplementary material for: Development of 1,2,3-Triazole-Based Sphingosine Kinase Inhibitors and Their Evaluation as Antiproliferative Agents
Source: Int J Mol Sci. 2017 Nov 5;18(11):2332. doi: 10.3390/ijms18112332 (PMC5713301; doi:10.3390/ijms18112332)
Supplement: Supplementary file 1 [file ijms-18-02332-s001.doc]

Supplementary Materials: Development of 1,2,3-Triazole-Based Sphingosine Kinase Inhibitors and Their Evaluation as Antiproliferative Agents

Angela Corvino, Roberta Rosa, Giuseppina Maria Incisivo, Ferdinando Fiorino, Francesco Frecentese, Elisa Magli , Elisa Perissutti , Irene Saccone, Vincenzo Santagada, Giuseppe Cirino, Maria Antonietta Riemma, Piero A. Temussi, Paola Ciciola, Roberto Bianco, Giuseppe Caliendo, Fiorentina Roviezzo and Beatrice Severino.

Figure S1. Percent of cell density of A549 NSCLC cells treated with different doses (0.5–5 µM) of the compound PF-543 for 3 days, as measured by 3-(4,5-dimethylthiazol-2-yl)-2,5-diphenyltetrazolium bromide (MTT) assay.
